# New insights into microstructure of neutron-irradiated tungsten

**DOI:** 10.1038/s41598-021-86746-6

**Published:** 2021-04-07

**Authors:** M. Dürrschnabel, M. Klimenkov, U. Jäntsch, M. Rieth, H. C. Schneider, D. Terentyev

**Affiliations:** 1 Institute for Applied Materials- Applied Materials Physics, Karlsruhe Institute of Technology (KIT), 76021 Karlsruhe, Germany; 2 Institute for Applied Materials - Materials- and Biomechanics, Karlsruhe Institute of Technology (KIT), 76021 Karlsruhe, Germany; 3 SCK CEN, Nuclear Materials Science Institute, Boeretang 200, 2400 Mol, Belgium

**Keywords:** Nuclear fusion and fission, Metals and alloys

## Abstract

The development of appropriate materials for fusion reactors that can sustain high neutron fluence at elevated temperatures remains a great challenge. Tungsten is one of the promising candidate materials for plasma-facing components of future fusion reactors, due to several favorable properties as for example a high melting point, a high sputtering resistivity, and a low coefficient of thermal expansion. The microstructural details of a tungsten sample with a 1.25 dpa (displacements per atom) damage dose after neutron irradiation at 800 °C were examined by transmission electron microscopy. Three types of radiation-induced defects were observed, analyzed and characterized: (1) voids with sizes ranging from 10 to 65 nm, (2) dislocation loops with a size of up to 10 nm and (3) W–Re–Os containing σ- and χ-type precipitates. The distribution of voids as well as the nature of the occurring dislocation loops were studied in detail. In addition, nano-chemical analyses revealed that the σ- and χ-type precipitates, which are sometimes attached to voids, are surrounded by a solid solution cloud enriched with Re. For the first time the crystallographic orientation relationship of the σ- and χ-phases to the W-matrix was specified. Furthermore, electron energy-loss spectroscopy could not unambiguously verify the presence of He within individual voids.

## Introduction

Tungsten (W) is the most cited material candidate for high-temperature vacuum or inert gas applications in power technology. Due to its favorable properties, such as the highest melting point in combination with high creep resistance, very high sputter resistance, a low coefficient of thermal expansion and a rather high thermal conductivity, it theoretically allows the raising of process temperatures into previously unknown temperature ranges. Besides renewable power generation methods, such as for example concentrated solar power, future fusion reactors will greatly benefit in particular by the use of W in plasma-facing structural parts. In the latter case, the material has to withstand very high operation temperatures as well as it has to tolerate a significant amount of radiation-induced damage^1–3^. A detailed experimental understanding of the neutron damage processes on the nanoscale, which is still limited at present time, is of utmost importance in the material qualification process.

Extensive microstructural characterizations of W have already been carried out to understand the microstructural response to neutron irradiation^3–8^. However, the covered irradiation doses and temperature range in these investigations are limited. Increasing both parameters to the conditions expected in a future fusion reactor will address still unsolved problems regarding the microstructure.

Basically, there are three mechanisms that may degrade the beneficial material properties of W during neutron irradiation: (1) Firstly, this includes the formation of lattice defects such as Frenkel pairs, interstitial and vacancy clusters and dislocation loops. (2) In addition, the formation of voids leads to swelling and radiation hardening that would restrict the lifetime of components beyond certain design limits. (3) The transmutation processes also lead to the formation of rhenium- (Re) or osmium-(Os) rich phases, while helium transmutation could stabilize voids. Both effects can lead to further significant embrittlement of W materials.

An additional issue arises with the experimental determination of the occurrence and dynamics of irradiation defects in material (fission) test reactors. They do not only depend on the neutron dose and temperature, but also on the transmutation rates, which themselves strongly hinge on the reactor type and the exact capsule position within the active reactor zone. As a consequence, each irradiation experiment has to be considered as a unique result, which paves the way for a more complete systematization and prediction of neutron radiation damage.

In contrast to technologically relevant neutron doses, irradiation in the sub-dpa range does not lead to a significant accumulation of Re or Os. In former studies, W–Re and W–Re–Os alloys were neutron-irradiated in order to simulate the influence of transmutation-induced elements on the microstructure and mechanical properties^9–11^. As was shown, the presence of Re influences the formation of voids and, thus, may contribute further to the hardening of the material. However, the obvious different behavior of Re and Os as solid solution in W opposed to their occurrence in form of transmuted elements is not yet fully understood. Therefore, in this work we focus not only on the comprehensive characterization of radiation induced loops and voids but also on the precipitation of Re- and Os-containing σ- and χ-phases. Moreover, as far as possible we examine and analyze the influence of grain boundaries (GBs) on formation of these defects. Finally, to maximize the output of the investigation on a set of unique irradiation samples, we make use of the recent progress in nanoanalytical methods implemented in transmission electron microscopy (TEM) such as for example the ChemiSTEM technology, high-speed DualEELS, and high-performance CCD cameras in order to understand the above mentioned neutron irradiation effects on technological W grade in more detail.

## Experimental

The investigated polycrystalline tungsten is of technical purity i.e. 99.7 wt%. This is technological material which is targeted for the application in ITER as armour for the divertor plasma facing components. The bulk material was supplied in a form of bar with dimensions 36 × 36 × 480 mm^3^, manufactured and stress-relieved by PLANSEE SE, Austria. The manufacturer provides the following list of impurities C, O, N, Fe, Ni, Si, and respectively the upper limit concentration of 30 (6), 20 (2), 5 (1), 30 (8), 20 (2) and 20 (1) µg/g, where the values in parentheses indicate typical values. This material has already been extensively studied in terms of mechanical properties, interaction with plasma and high heat flux loadings (see e.g.^12–14^).

The samples were irradiated by neutrons up to a damage dose of 1.25 dpa at 800 °C in the BR2 reactor located in Mol (Belgium). The irradiation was performed directly inside the fuel channel. The irradiation rig was made of a tick-wall pressurized tube which had a dual function: pressure-barrier and shielding to screen thermal neutrons. The temperature on the samples was actively controlled by the adjustment of helium pressure inside the rig. Helium environment also prevented oxidation under high temperature exposure. The total irradiation time was 143 days and the neutron flux was 5 10^14^ (E > 0.1 meV), 2 10^14^ (E > 1 meV) and 4.2 10^14^ (E < 0.1 meV) n/cm^2^/s^15^. The neutron flux was calculated using MCNPX 2.7.0^16^ code as well as confirmed by dosimetry measurements using Fe and Nb dosimeters, applied to measure the fast neutron fluence (> 1 meV). The dpa cross sections for W have been prepared from the JENDL4 file (MT444) for the threshold displacement energy of 55 eV.

The calculation of the transmutation product activities, as well as their evolution in time, was performed using the nuclide inventory code FISPACT-II^17^ with cross section data from both TENDL-2017^18^ and EAF-2010 library^19^. Alternative calculations of the transmutation inventory was also performed using ALEPH code^20^ by assessing the propagation of the neutron spectrum from the fuel channel through to the stainless steel wall and then inside the sample. Both of the approaches resulted in a very similar output. The transmutation induced content of Re and Os was determined to be ~ 2 at.% and 0.2 at.%, respectively. Without the stainless steel shielding, the rhenium transmutation was estimated to be ~ 4 at.% per dpa unit. More details about the irradiation device and results on mechanical tests could be found in Refs.^15,21^ Following the above described calculations, the generation of helium and hydrogen at 1 dpa is expected to be 6 * 10^−3^ appm and 1.8 * 10^−3^ appm, respectively.

Since the material is radioactive, it is advantageous to use a focused ion beam machine (FIB, FEI Scios) for specimen preparation to (1) limit the radiation exposure and (2) the specimen preparation in a predefined area. Thin foils for TEM investigations were prepared using the FIB technique in Fusion Materials Laboratory (FML) at KIT. The thin lamellae were attached to a molybdenum grid. After preparation the lamella was flash polished in 1% NaOH water solution using method described in the ref.^22^.

TEM analysis was performed in a Thermofisher Talos F200X scanning transmission electron microscope (STEM) equipped with four energy-dispersive X-ray (EDX) detectors. STEM-EDX maps were acquired in the Velox software using 512 × 512 pixels and a spectral dispersion of 5 eV. The EDX detector resolution is specified by the manufacturer as ≤ 136 eV at Mn-K_α_. At the W-L_α_ (E = 8.396 keV) the energy resolution has a value of about 150–160 eV, which is sufficient to separate the W-L_α_, Re-L_α_ and Os-L_α_ X-ray lines. Quantification of STEM-EDX data was done using the Cliff-Lorimer k-factor method. The TEM images and selected area diffraction pattern (SAED) were acquired by using a Thermofisher Ceta 16 M CCD camera. The STEM-EELS data was acquired using a convergence angle of 10.5 mrad and a collection angle of 14.1 mrad. Furthermore, the extraction voltage was reduced such that the final energy resolution was about 0.7 eV.

The diffractograms obtained from high-resolution phase contrast images were fitted using JEMS software package^23^. The crystallographic structures of the individual phases that were used as input are summarized in Table 1. In case of the WOs_3_ χ-phase no structural model was available, therefore, we used WRe_3_ χ-phase and replaced Re by Os, which should be feasible since atomic radii of both atoms differ only by a few picometers^24^.

**Table 1:** Summary of crystal structures used for JEMS modelling.

Phase	ICSD No	Space group	Lattice
W	43421	Im-3 m	Cubic, bcc, a = 3.17 Å
σ-WOs_2_	150547	P42/mnm	Tetragonal a = b = 9.43 Å c = 4.99 Å
χ-WOs_3_	650196*	I-43 m	Cubic, a = 9.60 Å

*ICSD number belongs to the WRe_3_ structure.

Data post processing was performed using the HyperSpy software package^25^ and Gatan Digital Micrograph. Independent component analysis (ICA) was performed via the FastICA algorithm on the results of a principal component analysis (PCA) as implemented in the Hyperspy package. The data was normalized during PCA with respect to Poisson statistics. The STEM-EDX data was binned by a factor of two in spectral direction prior to PCA analysis.

The analysis of the Burgers vector of dislocation loops includes dark field imaging under defined orientation of the g-vector near the [110] zone axis. The visibility criteria for dislocation loops with b½ < 111 > and < 100 > are given in the Table 2.

**Table 2:**
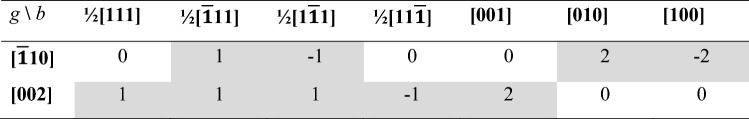
Visibility criterion for dislocation loops in diffraction contrast microscopy.

## Results

The microstructure of “as-delivered” material was detailed characterized and published in refs.^15,26^. The investigated tungsten shows an average grain structure with a size of 88 µm. Detailed TEM analyses revealed the occurrence of three types of irradiation defects: (1) voids with a size ranging from 10 to 65 nm, (2) "black dots" and dislocation loops with a size of up to 10 nm and (3) W–Re–Os containing σ- and χ-type precipitates. A detailed TEM characterization of all three defect types is presented in the next subsections. From the previous studies of neutron irradiated W and beryllium it is well known^6,27^ that the accumulation of irradiation defects near GB and in the grain interior differs, i.e. so-called denuded zone is formed close to the GB, which is depleted by or even completely free of any void. Therefore, it is important to examine the formation of radiation-induced defects in the two areas, i.e. grain interior and GB.

### Distribution of voids

**Figure 1:**
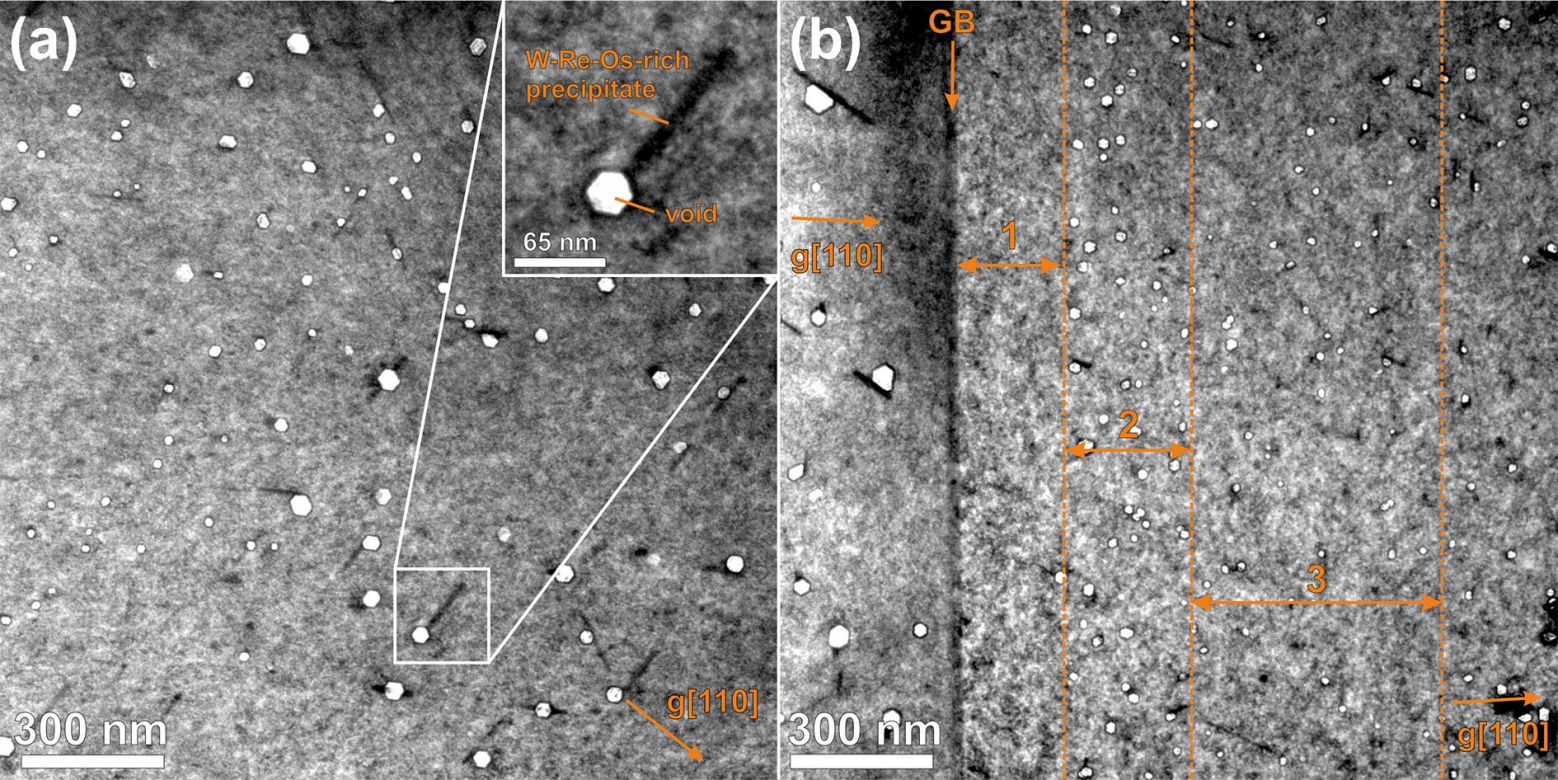
Bright-field TEM images acquired from neutron-irradiated W. (**a**) A representative area of a grain interior with voids (bright spots) and Re–Os-rich precipitates (dark strips). The inset shows a magnified view of a individual void attached to a W–Re–Os-rich precipitate. (**b**) A representative GB region. The numbered areas are (1) the void denuded zone, (2) void-peak zone and (3) the second denuded zone.

Figure 1 shows TEM bright-field images of a representative grain interior (Fig. 1a) and a depicts a representative GB region (Fig. 1b). The images were acquired with orientation near [133] zone axis. This GB is a twist boundary with a total misorientation of 9°. The twist occurs around [011] axis which is perpendicular (with a misorientation of 3°) to the bounding plane. The voids that were generated by neutron irradiation appear in bright contrast. This was exploited to determine their size distribution as well as their number density. The local sample thickness needed to determine the number density was estimated via quantification of EDX spectra. In the grain interior (Fig. 1a) voids with a maximum size of 65 nm and a number density of (4.3 ± 1.2) 10^20^ m^−3^ were found. In addition, the voids located in the grain interior are more or less homogeneously distributed. The inset in Fig. 1a proves that the voids are also faceted, i.e. the facets are of (110)-type. As can be seen in Fig. 1a, some voids are attached to W–Re–Os-rich precipitates having a darker contrast than the surrounding W matrix. A detailed analysis of these Re–Os-rich precipitates is carried out in “Segregation of transmutation products” section.

At the GB the spatial distribution of voids is markedly different than in the grain interior as can be seen by comparing features on Fig. 1a,b. Three different well distinguishable zones are found: the (1) void denuded zone, (2) void-peak zone and (3) the second denuded zone. The void denuded zone is directly attached to the GB in the present case, it has a width of about 200 nm being completely free of voids (see Table 3). The “void peak zone” has a width of about 300 nm. The notion “void peak zone” is due to the increased number density of voids, which is about twice as high as that determined in the grain interior as can be seen in Table 3. This zone is followed by the second denuded zone, which is about 500 nm in width. Here the void number density considerably decreases to about half of the value measured in the grain interior (see Table 3). The homogeneous distribution of voids, which is typical for the grain interior, is observed at 1 µm away from GB.

**Table 3:** Quantitative and statistical data of voids obtained near the GBs separately for different zones.

Numbered in Fig. 1	Designation	Location to GB /nm	Void number density/× 10^20^ m^−3^	Average void size /nm	Void swelling /%	Average loop size/nm	Loop number density/× 10^23^ m^−3^
1	Void and precipitates denuded zone	From GB to 200 nm	–	–	–		
2	Void peak zone	from 200 to 500 nm	8.0 ± 2.5	24	~ 0.004		
3	Second denuded zone	From 500 nm to 1 µm	1.9 ± 0.5	22	–		
–	Grain interior	> 1 µm	4.3 ± 1.2	31	~ 0.02	5.2 ± 1	4.1 ± 1.5

**Figure 2:**
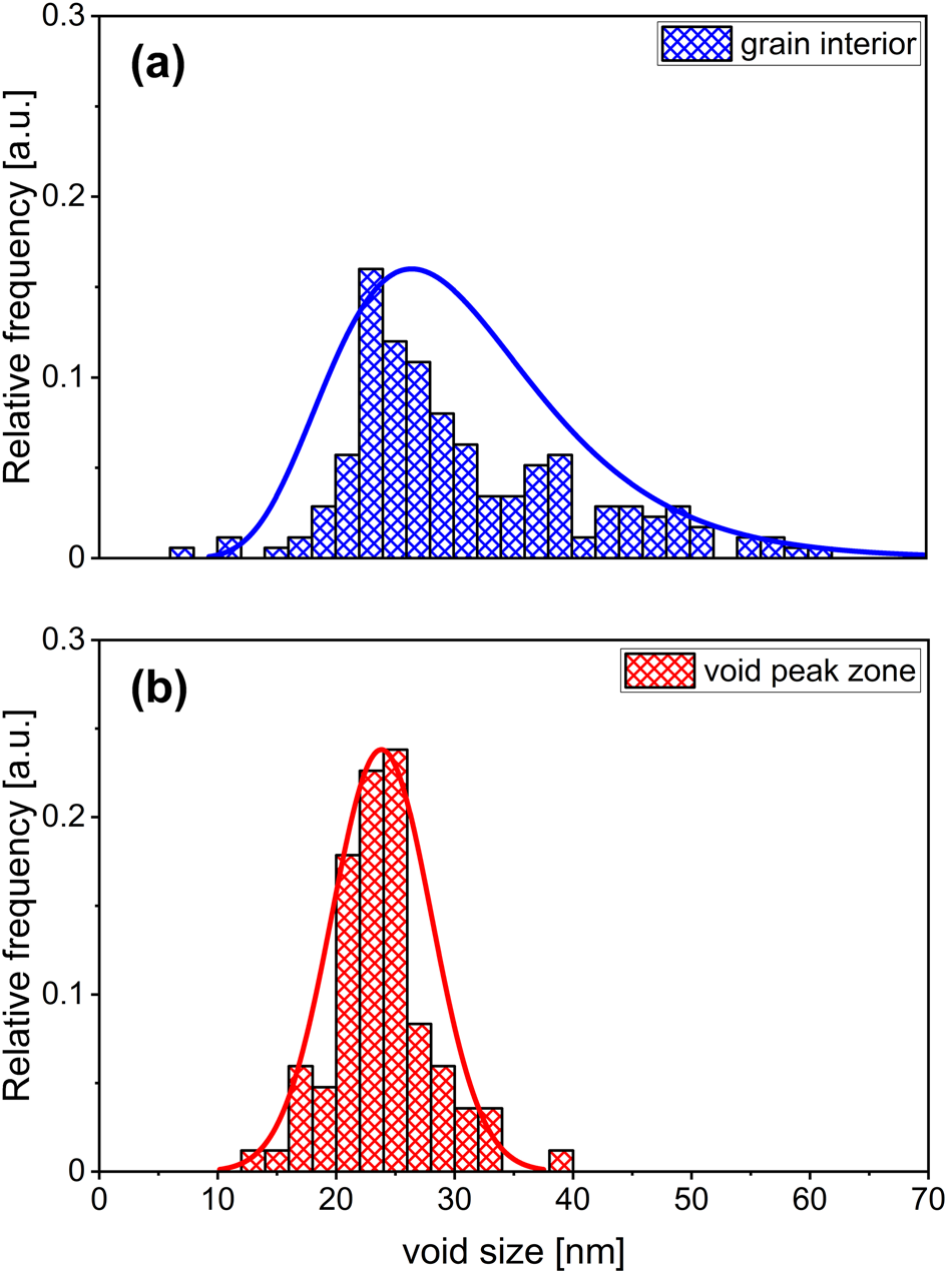
Size distribution histograms of the voids registered in the (**a**) grain interior and (**b**) void peak zone.

The size distribution histograms for voids registered in the grain interior (blue bars) and in the void peak zone (red bars) are shown in Fig. 2. In the grain interior the voids have a log-normal-type size distribution as can be seen in Fig. 2a, whereas in the void peak zone voids have Gaussian-like distribution profile. In the grain interior the void sizes are ranging from 8 to 60 nm and have an average size of 31 nm. In the void peak zone, the void sizes are ranging from 14 to 39 nm with an average size of 24 nm.

### Dislocation loops

**Figure 3:**
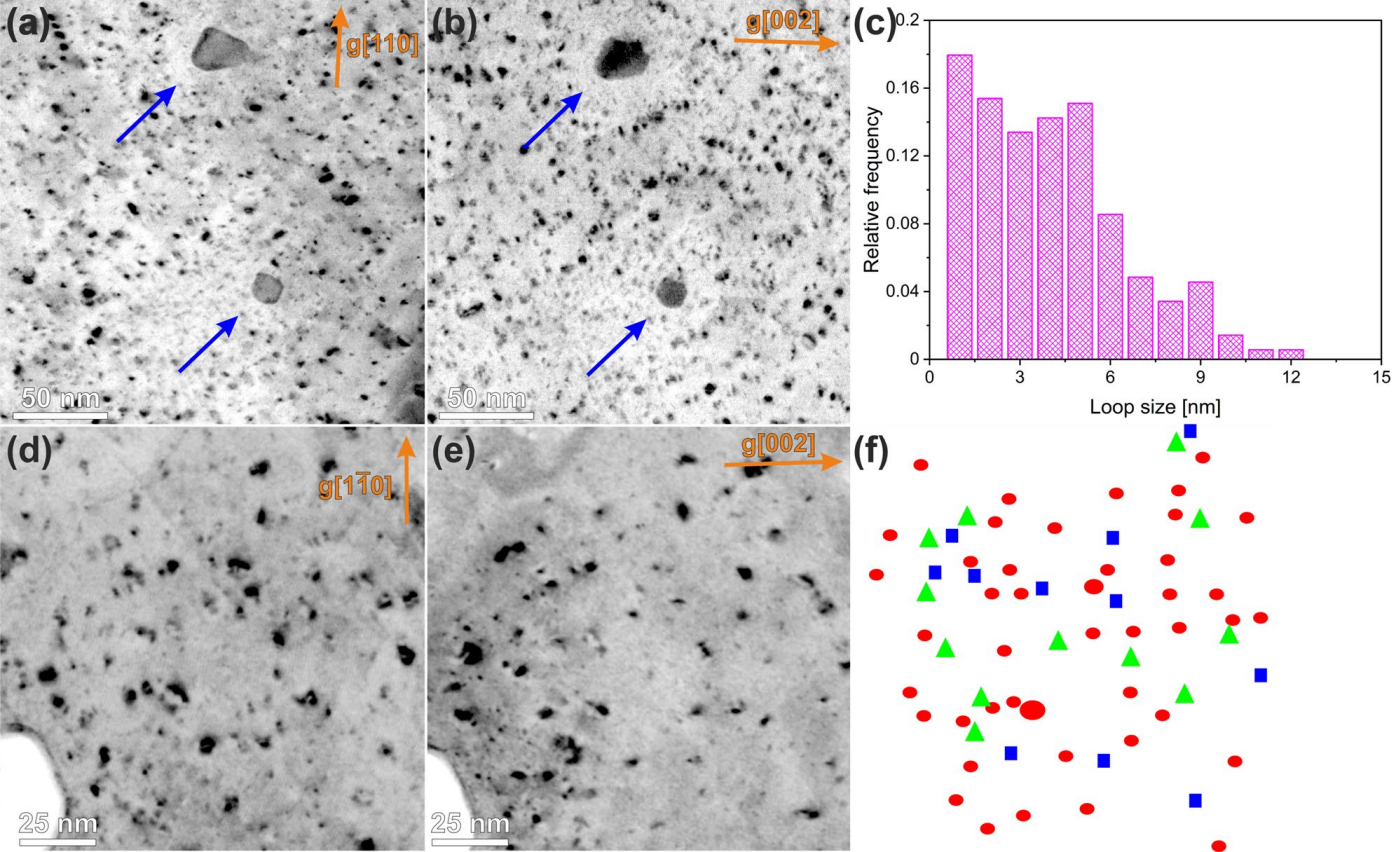
Visualisation of dislocation loops and black dots using reverse contrast dark field (DF) imaging. DF images were obtained with g[110] (**a**, **d**) and g[002] (**b**, **e**) g-vectors near the [110] zone-axis. The size distribution histogram of the dislocation loops is shown in (**c**). The loops with *b*½〈111〉 and b〈100〉 Burgers vectors are imaged in the part (**f**) with red circles and blue squares, respectively. The loops with undefined Burgers vector are denoted by green triangles.

In addition to the voids, dislocation loops are also generated in considerable amount during neutron irradiation, which are visible mainly as small featureless black dots and circular loops. Figure 3 visualizes typical neutron-irradiation defects by reverse contrast dark-field (DF) imaging with defined g-vectors. Figure 3a,b show a general view of dislocation loops, which were imaged with different g-vectors near the [110] zone-axis. The contrast in such images is uniform and less sensitive to local variations of sample thickness. These images are the basis for the loop size histogram shown in Fig. 3c. The average size of all loops was measured to be 4.5 nm, with a significant fraction of defects being featureless black dots less than 6 nm in size. The calculation of the defect number density normalized to the local layer thickness leads to a value of about (4.0 ± 1.5) 10^23^ m^−3^. The uncertainty in the determination of local sample thickness is the reason for the 30% error of this value. The areas of about 10–20 nm around the precipitates, which are marked with blue arrows in Fig. 3a,b, are almost free of dislocation loops. As will be shown in the next sections, the areas around the precipitates also exhibit increased Re and Os local concentration, which presumably affect the formation of the loops^4,28^.

The high number density as well as the small size of the defects hamper the determination of the Burger’s vectors in Fig. 3a,b applying the visibility criteria or identification habit plane as presented in^29^. The value given in Table 3 was taken as the average of the two methods.

The ${N}_{\langle 100\rangle }/{N}_{1/2\langle 111\rangle }$ ratio was obtained by the method described in ref.^30^ using the visible number densities for g[−110] and g[002] vectors. The result shows that (25 ± 20)% of the defects have a $b\langle 100\rangle$ and (75 ± 20)% a $b{1/2}\langle 111\rangle$ Burgers vector. To minimize the above-mentioned problems and to improve the statistics for the loops with a size of less than 3 nm, the large-magnification reverse contrast DF images were acquired from a 40 nm thick area near the [110] zone axis (Fig. 3d,e). The Burgers vector of the defects was analyzed according to the visibility criteria and the possible habit plane (Table 2). The results of this analysis are shown with geometric shapes of different colors in Fig. 3f. These methods show that 15% of the defects have a $b\langle 100\rangle$ and 85% a $b{1/2}\langle 111\rangle$ Burger’s vector. This finding is in good agreement with the results found on the basis of the number density. The value given in Table 3 was taken as the average of the two methods.

### Segregation of transmutation products

**Figure 4:**
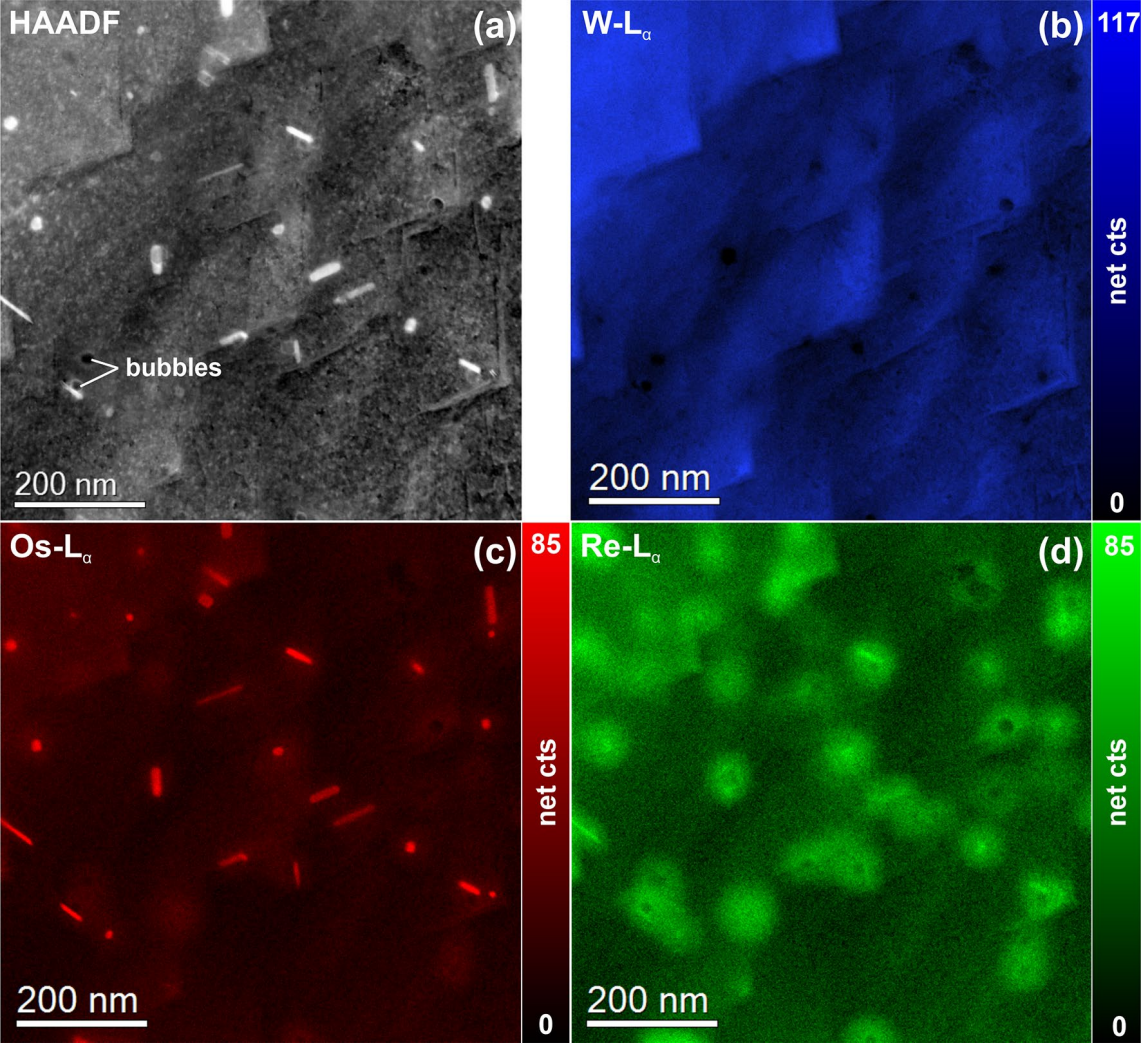
STEM-EDX spectrum image of a representative region in a neutron-irradiated W specimen. (**a**) STEM-HAADF image. Elemental maps of (**b**) W, (**c**) Os, and (**d**) Re display the location of elements with respect to the sample morphology. The tile-like structure in the HAADF image and the W map is surface topography due to flash polishing.

Figure 4 shows a STEM-EDX spectrum image of a representative region in a neutron-irradiated W specimen. The STEM-HAADF image in Fig. 4 reveals that the precipitates are composed of atoms with Z number larger than the W matrix. It also shows that the sample has a surface topography due to selective etching during flash polishing, which is confirmed by the W-L map, see Fig. 4b. The bright precipitates in the HAADF image are determined to be Os-rich particles as can be seen in the Os-L map, see Fig. 4c. In addition, Fig. 4c points out that two types of precipitates co-exist: (1) rod-shaped ones with a size is 50–70 nm and (2) spherical or polyhedral-shaped ones with a size of 15–20 nm. Furthermore, it is found that a Re-rich “cloud-like” phase is present around the Os-rich precipitates (see Re map in Fig. 4d). The STEM-HAADF image in Fig. 4a exhibits no contrast change which indicates that the Re-enriched region has a coherent to matrix structure. The size of these Re-rich “clouds” depends on the size of the Os-rich core as well as on the density of the precipitates (i.e. in case of closely spaced Os-rich particles the respective Re-clouds overlap) and typically it is about 100 nm. Furthermore, some voids with attached to the Os-rich precipitates were observed within the W matrix.

**Figure 5:**
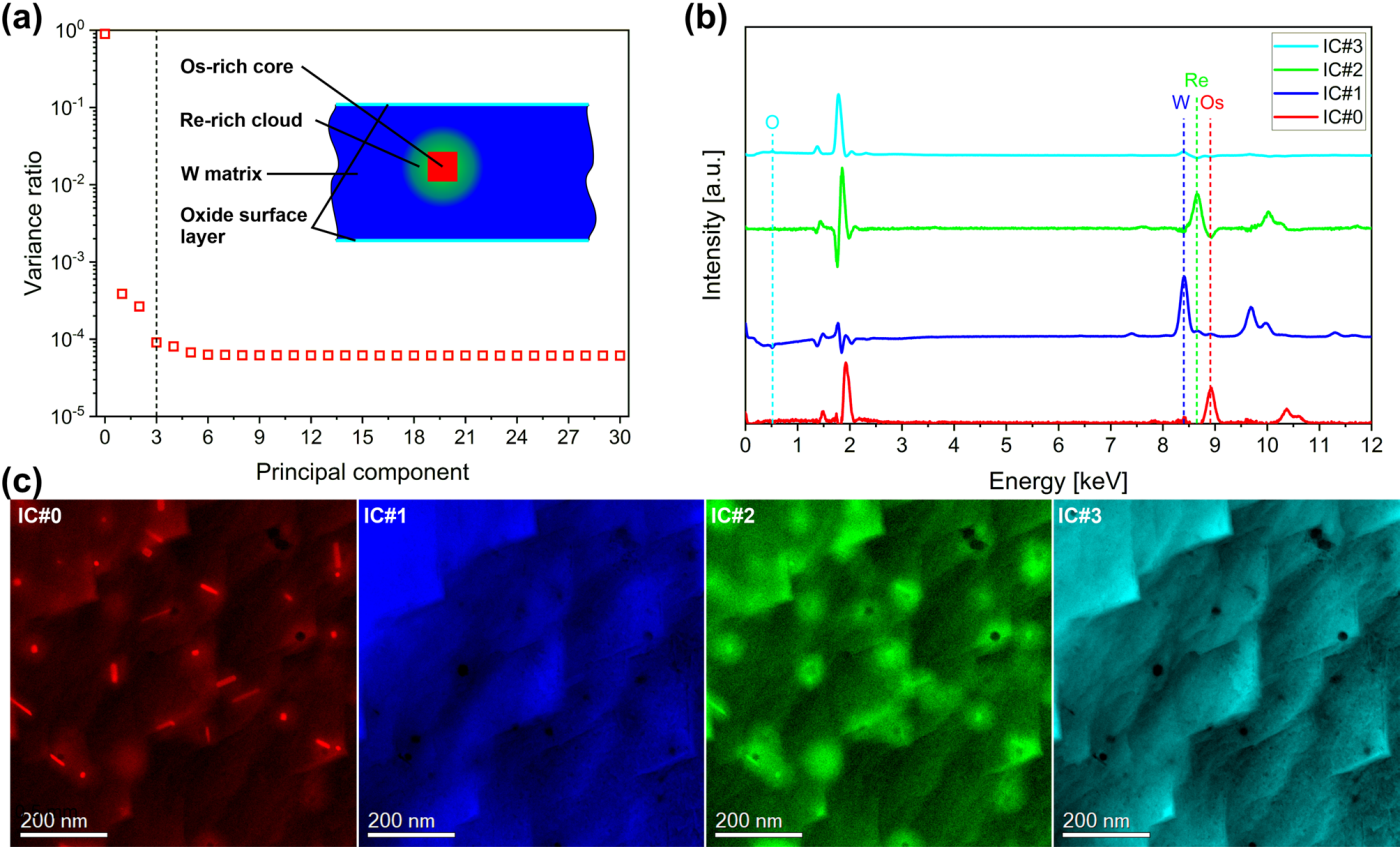
Results of a blind source separation (BSS) by PCA and ICA of the STEM-EDX data presented in Fig. 6. (**a**) Screen plot of the first 30 principal components (the dashed line indicates the number of independent components). The inset shows a schematic cross-sectional view of the sample for illustration purposes. (**b**) Corresponding independent component spectra. (**c**) Independent component maps showing the presence of Os-rich cores (IC#0), a W matrix (IC#1), a Re-rich cloud around the Os-rich cores (IC#2), and an oxide surface layer (IC#3), which is most probably due to the sample preparation.

In order to understand the phases and their distribution in more detail, an ICA was performed on the STEM-EDX data presented in Fig. 4. The results are shown Fig. 5. Figure 5a illustrates that the first 4 principal components have a significantly larger variance than the remaining components. The inset shows a schematic representation of the sample that was derived by analyzing the individual independent component spectra (Fig. 5b) and respective maps (Fig. 7c). IC#0 corresponds to the Os-rich precipitates that are surrounded by Re-rich clouds (IC#2). Both are embedded in the W matrix (IC#1). No significant elemental intermixture between the three elements was observed here. The fourth independent component was a thin oxide surface layer that originated most probably from sample preparation and could not be avoided.

**Figure 6:**
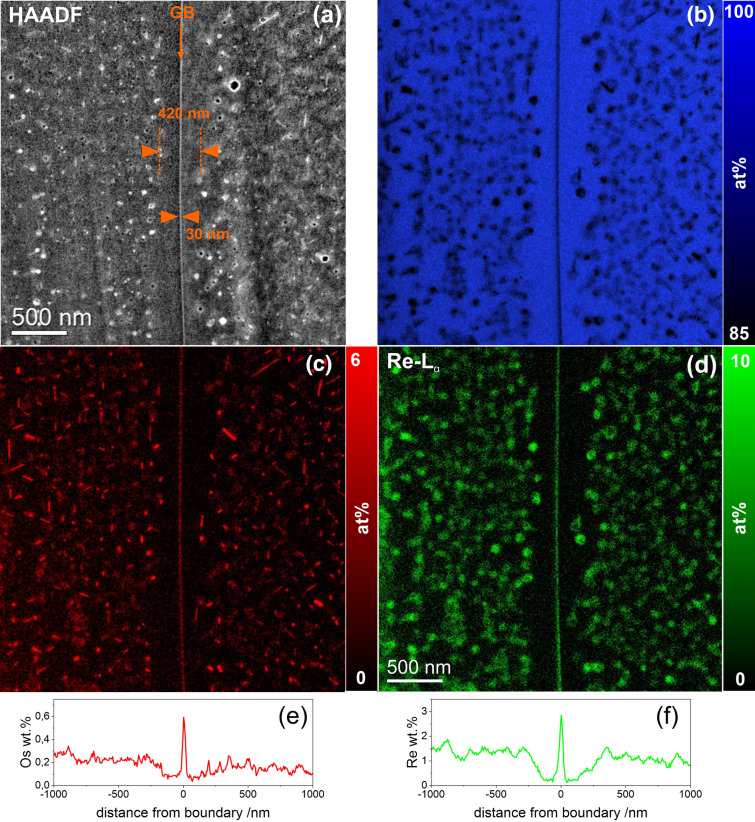
STEM-EDX mapping near GB. (**a**) STEM-HAADF image with marked position of GB. Elemental maps of (**b**) W, (**c**) Os, and (**d**) Re display the location of elements with respect to the sample morphology. The profiles in parts (**e**) and (**f**) indicate the distribution of the Os and Re concentration near the GB.

The STEM HAADF image in the upper part of Fig. 6a shows a representative sample region containing a GB that was shown in Fig. 1b (marked as GB). In addition, it is evident from the image that in each W grain there is a denuded zone present that has a width of about 210 nm. Further into the grain interior the situation is as described in Fig. 6. Furthermore, it was found that in the center of the GB there is an enrichment of Re (3.6 wt%) and Os (0.7 wt%) that has a width of about 30 nm, which can be observed in the elemental maps (Fig. 6b–d) and in the line profiles (Fig. 6e–f). The Re and Os intensity profiles were obtained by integrating of ~ 1 µm wide strips across GB. The small differences in the parallel run of the GB reduce the displayed Re and Os concentration in the profiles. Taking into account these facts, the specified values were measured exactly in the middle of the GB.

### HRTEM analysis of precipitates

**Figure 7:**
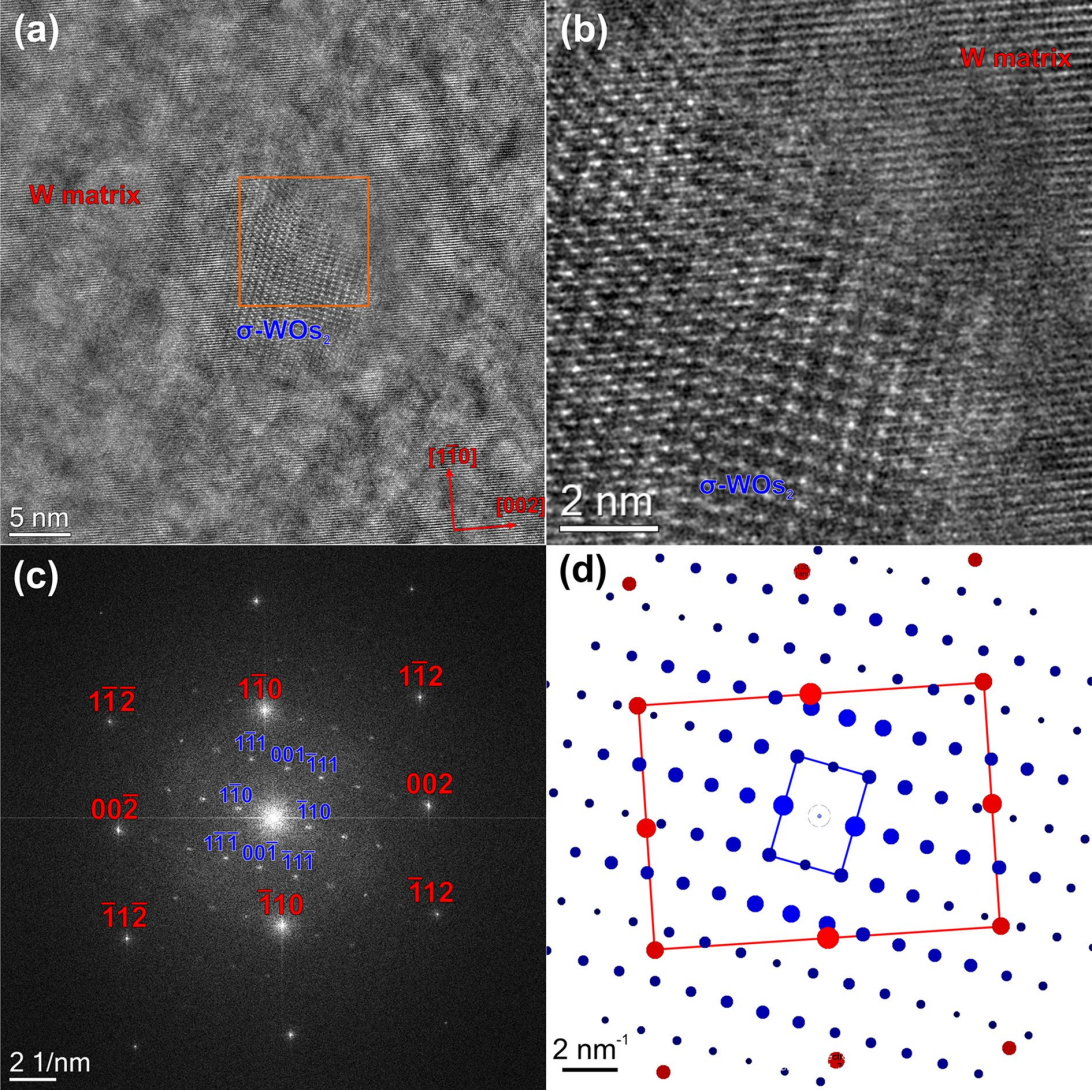
(**a**) High-resolution phase contrast image of a spherical-shaped Os-rich precipitate, which is identified as the WOs_2_ σ-phase. Magnified view (**b**) and diffractogram (FFT) (**c**) of the region delimited by the orange square in (**a**). (**d**) Simulated model of the diffractogram.

**Figure 8:**
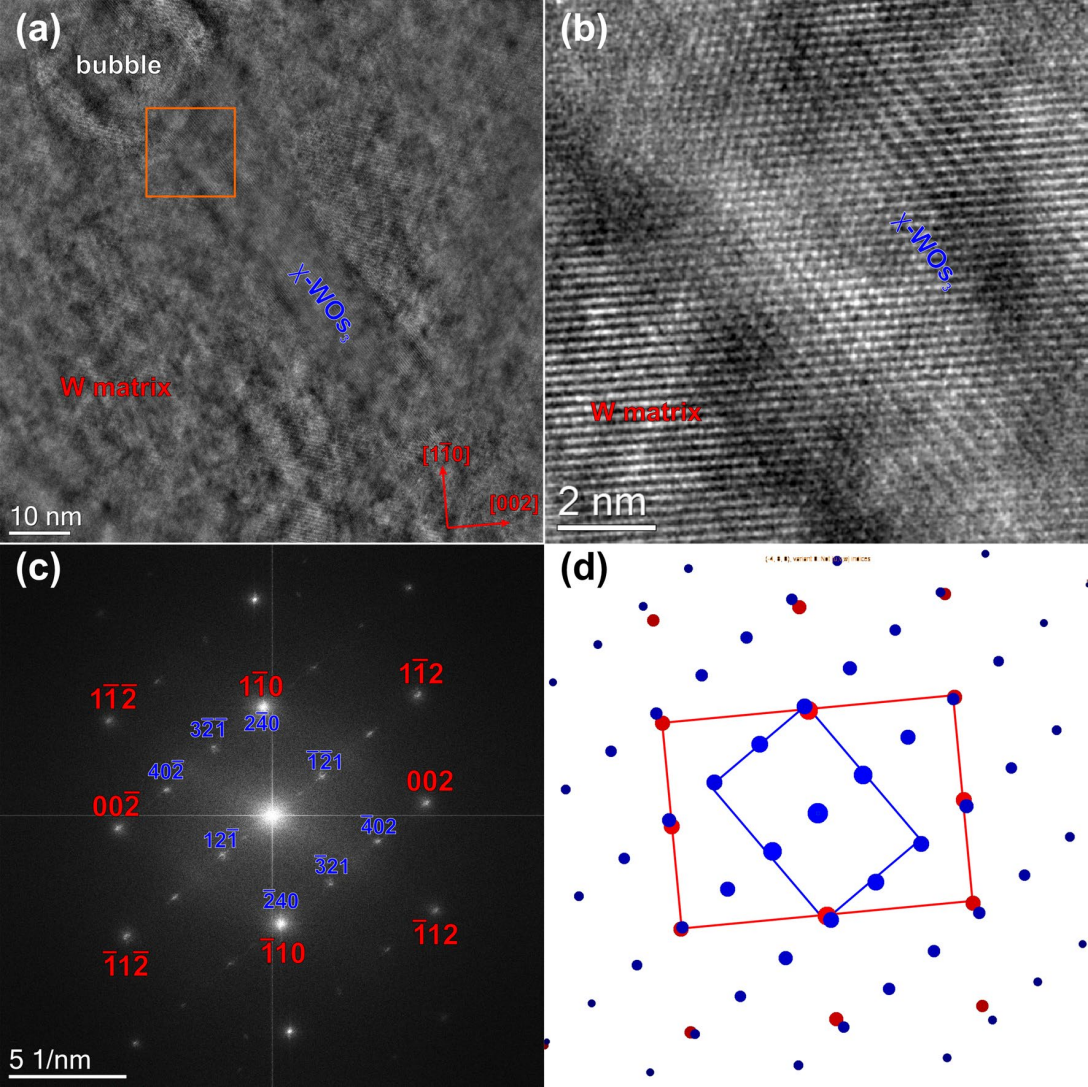
(**a**) High-resolution phase contrast image of a rod-shaped Os-rich precipitate, which is identified as the WOs_3_ χ-Phase. Magnified view (**b**) and diffractogram (FFT) (**c**) of the region delimited by the orange square in (**a**). Simulated model of the diffractogram (**d**).

**Figure 9:**
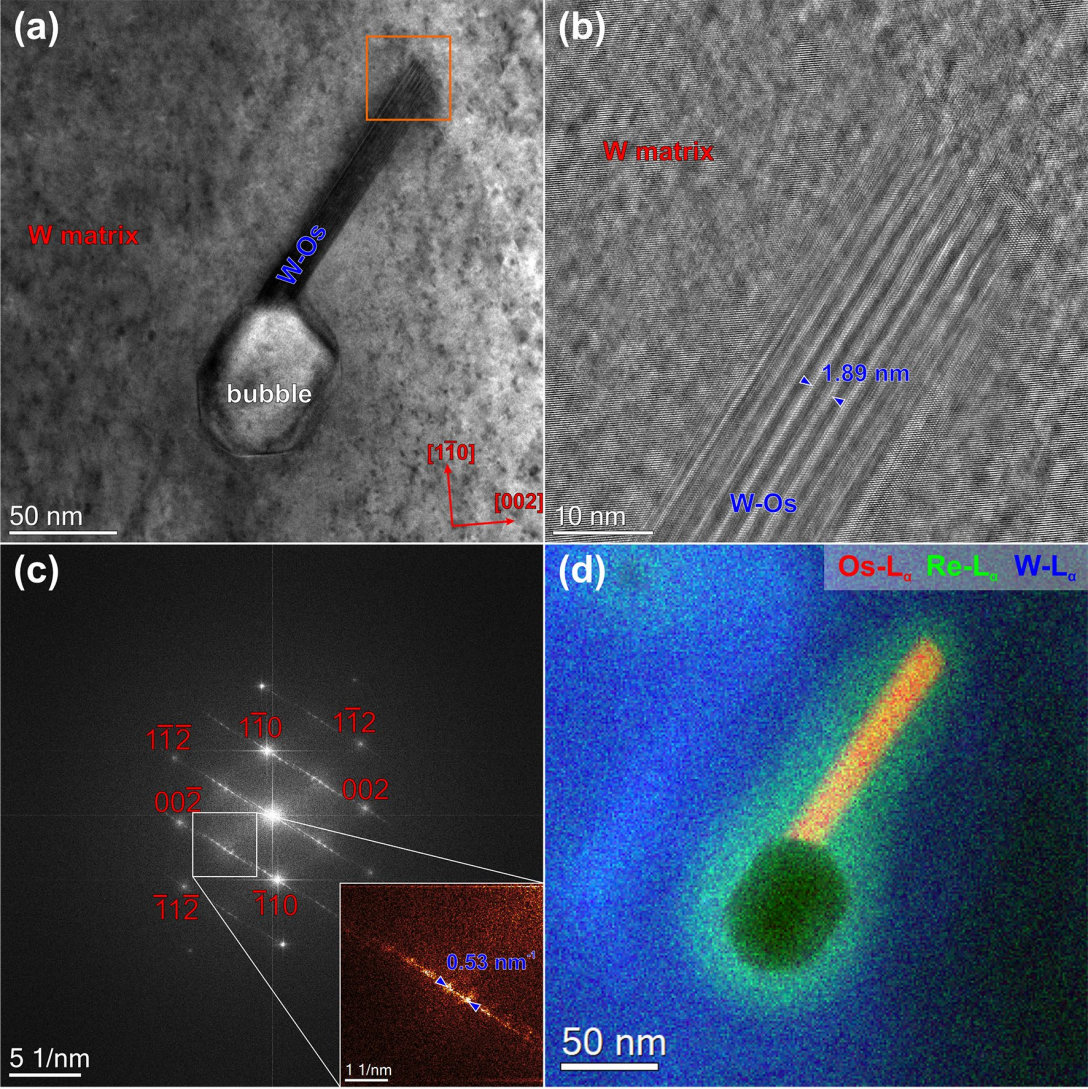
(**a**) TEM bright-field image of a faceted void attached to a W–Os rod. (**b**) High-resolution phase contrast image of the W–Os phase exhibiting Moiré fringes in the Os-rich part. (**c**) Diffractogram (FFT) of (**b**). (**d**) Composite STEM-EDX elemental map of the same sample region.

High-resolution phase contrast images are acquired in order to further characterize the Os-rich precipitates as can be seen in Figs. 7, 8, and 9. In these three figures, no structural difference between the pure W matrix and the Re-enriched cloud around the Os-rich precipitates are observed. Therefore, the region outside the Os-rich precipitates is labeled as W matrix in these figures. Figure 7a shows a high-resolution phase contrast image of a spherically shaped Os-rich phase embedded in the W matrix. The W matrix is positioned to have a [110] zone-axis orientation. Figure 7b presents a magnified view of the region delimited by the orange square in (a). It is evident that the precipitate has the different crystalline structure compared to the matrix. However, without knowing the exact sample thickness the assignment of atomic positions in the precipitate is ambiguous due to the contrast reversals occurring for this particular imaging mode. Nevertheless, the fast Fourier transform (FFT) in Fig. 7c calculated from Fig. 7a sheds some light on the crystallographic structure of the precipitate and its crystallographic relationship to the W host matrix. The spots corresponding to the W matrix are indexed in red color and the ones originating from the precipitate are indexed in blue. For indexing the precipitate, we used the crystal structure of the WOs_2_ σ-phase (ICSD No. 150547), which is tetragonal. Furthermore, diffraction pattern calculations were carried out using the JEMS software (Fig. 7d) in order to highlight the orientation relationship, i.e. (1–10)_W_ || (1–12)_σ_, (002)_W_ || (−441)_σ_ and [110]_W_ || [110]_σ_.

Figure 8a shows a high-resolution phase contrast image of a rod-shaped Os-rich precipitate, which is attached to a void. Figure 8b is a magnified view of the region delimited by the orange square in (a). The situation is less evident here than in Fig. 8c, because there is only a slight change of the contrast. However, the diffractogram in Fig. 8c reveals the presence of two different crystal lattices, i.e. the matrix oriented in [110] zone-axis (red indices) and the precipitate (blue indices). For indexing the precipitate, we used the crystalline structure of the WRe_3_ χ-phase as model structure (ICSD No. 650196) since no structure of a W–Os χ-phase is reported in the ICSD database. The WRe_3_ χ-phase has a body-centered cubic structure just as W. We therefore just replaced Re by Os, which should not remarkably affect the structure since the atomic radii of these atoms differ only by a few picometers^24^ and carried out diffraction pattern simulations by the JEMS software^23^. However, to provide more accurate results, the structure needs to be relaxed e.g. by applying density functional methods. The result of our simulation is shown in Fig. 8d. The orientation relationship in this case was determined as (1–10)_W_ || (2–40)_χ_, (002)_W_ || (−5–32)_χ_ and [110]_W_ || [214]_χ_.

Figure 9a shows a rod-shaped Os-rich precipitate attached to a void of about 75 nm in size. The void surface is facetted, i.e. {110}, {002}, and {112}-type facets can be observed. The Os-rich precipitate is attached to a {110}-type facet and seems to grow along a ${\langle 112\rangle }_{W}$-type direction. However, as can be seen in Fig. 9b, there is Moiré contrast present that indicates that the lattice of the precipitate is shifted, rotated or both with respect to the matrix lattice. The Moiré fringes have a distance of about 1.9 nm, which is also reflected in the diffractogram (see inset). Furthermore, the diffractogram suggests that the precipitate is a χ-type because the {111}-type planes seem to be aligned, since the distance of (111)_χ_ is about 1/3 of (111)_W_. Careful analyses of Fig. 9a points out that the precipitate is inclined with respect to the image plane, i.e. clear Moiré contrast in the upper part vs. no Moiré contrast in the lower part (close to the void) of the precipitate. Figure 9d presents a composite image that shows the elemental distribution of Os in red, the Re distribution in green and the W distribution in blue colours.

### EELS analysis of voids

**Figure 10:**
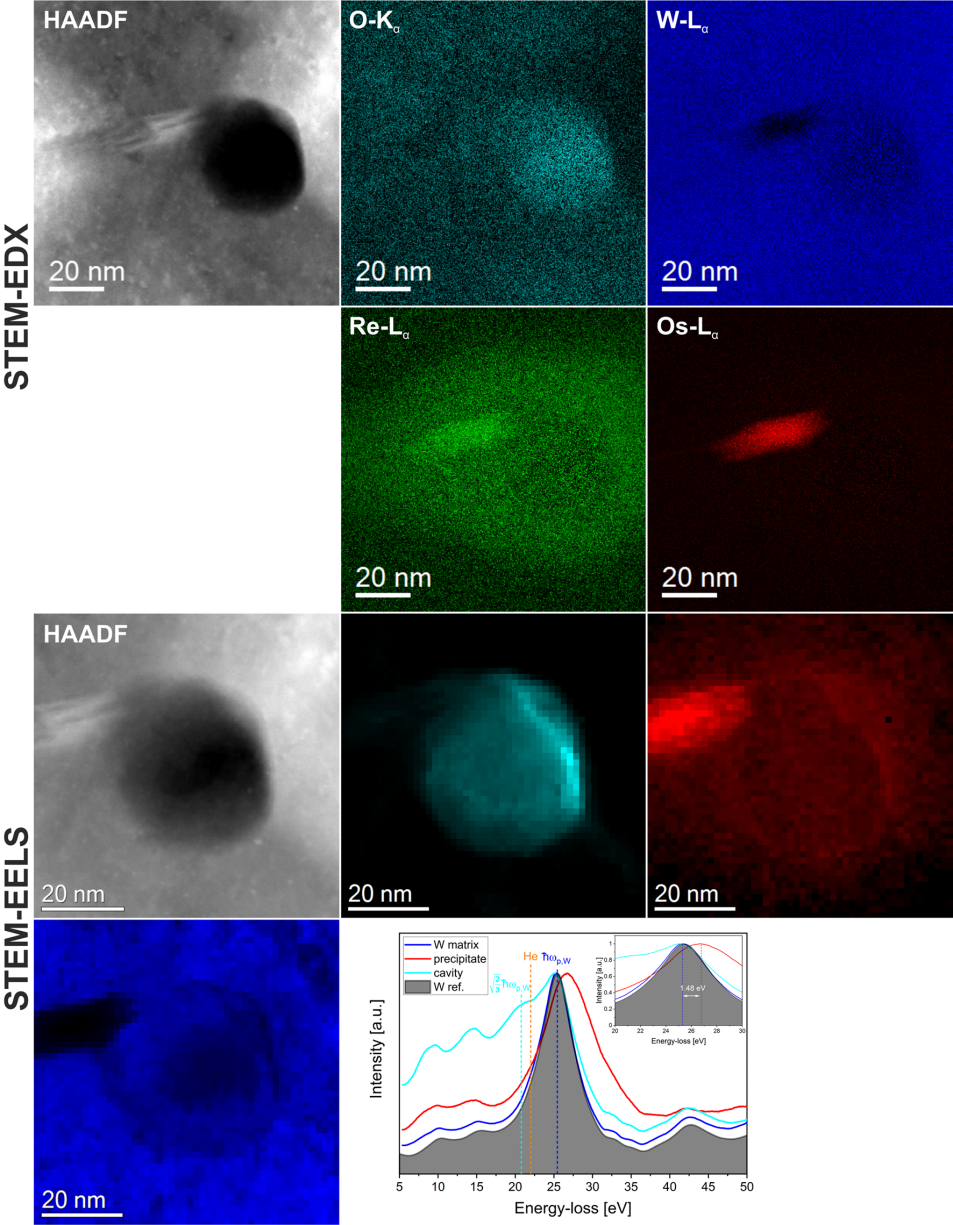
Combined STEM-EDX and low-loss STEM-EELS measurement of a individual void. The intensities of the STEM-EELS maps was obtained by NLLS fitting of the peaks located in the W plasmon peak area.

Figure 10 presents a combined STEM-EDX/STEM-EELS measurements performed for of a individual void. The STEM-EDX in the upper part shows that the void is attached to a Re–Os-rich precipitate and is also surrounded by a Re-rich cloud. In addition, oxygen was fund to be present within the void. The STEM-EELS measurements presented in the lower part are performed to determine whether He is present in the void or not. An analysis of the acquired low-loss EELS data (shown on the right side in the lowest row) revealed that the bulk W spectrum is similar to a spectrum acquired from an unirradiated W sheet. Furthermore, it shows that the plasmon peak of the precipitate is shifted to higher energy-losses by 1.48 eV with respect to the W matrix one. An additional peak (indicated by the orange dashed line) is present at an energy loss of about 20–21 eV in the void area, the energy corresponding to the position of the He edge.

## Discussion

The radiation-induced dislocation loops or defects, which are visible as structureless black dots a few nanometers in size, have an average size of 5 nm and a number density of 4 10^23^ m^−3^ (Fig. 3, Table 3). Detailed information about the quantitative parameters of these defects and an understanding of the nature of these defects are necessary to explain e.g. radiation-induced embrittlement. As has been shown in several studies, the size and number density of dislocation loops do not vary significantly over a wide range of irradiation temperatures and damage doses^8,31,32^. The average size of 5 nm is consistent with previously reported data for neutron-irradiated tungsten, where it typically ranges from 1.2 nm to 8.0 nm^8^. The formation of larger loops has rarely been reported in the scientific literature, e.g. Williams et.^28^ showed the formation of 150 nm loops in a W–Re alloy after neutron irradiation at 1500 °C. The measured number density is considerably higher compared to the literature, where its value varies between 1 10^22^ m^−3^ and 1 10^23^ m^−3^. This could reflect successful flash polishing procedure, which allows one an accurate detection of ~ 1–2 nm loops.

The dislocation loops were observed in neutron irradiated W at an irradiation dose of 0.03 dpa/ 90°C^8^ and usually appear up to 600 °C irradiation temperatures^11,32–34^. However, two works, which are consistent with our results, showed its presence up to 800°C^8,34^. The recent temperature-damage diagrams covers the irradiation temperature range from 100 to 900 °C and damage doses of up to 2.0 dpa^35^. The damage structures were classified into three types: “loops”, “loops and voids” and “precipitates and voids”. It shows that at temperatures exceeding 500 °C dislocation loops occur in combination with voids for doses below 0.6–0.7 dpa, whereas for doses larger than 1.0 dpa the formation of loops was not reported. The critical aspect is that the diagram is based on a limited number of measurements, suggesting that the areas of the sections are not well defined and the occurrence of damage may deviate. In particular, this explains the deviation from our results concerning dislocation loops which show their existence at 1.25 dpa/800 °C. This indicates that the areas for the formation of dislocation loops should be modified.

In general, the observation of dislocation loops with TEM is a challenging task. By far not all publications contain the micrographs on which the loops could be identified. Due to the radioactive nature and small dimensions, most of TEM specimens have to be prepared by FIB, which itself causes severe surface damage hindering a doubtless identification of the loops. To remove the damaged layer, a low-energy ion cleaning or flash polishing have to be used. This procedure, however, is not successful in all cases so that the detection of small-sized dislocation loops (< 5 nm) remains a challenge for an accurate quantification.

Burgers vector analysis of dislocation loops have only been reported for ion irradiated materials. In most cases the formation the loops in W show b½ < 111 > Burgers vector^36–38^, however, the presence of a small amount (4%—6%) of b < 100 > loops was reported^39^. These results are consistent with our analysis, which shows that 85% of the loops have a b½ < 111 > Burgers vector and only a small fraction of b < 100 > loops was observed. In the few publications where the nature of dislocation loops has been investigated experimentally, it has been shown that the loops in W have mostly interstitial nature^34,40^. The observed formation of vacancy loops of < 100 > type was reported only in the few cases and only with^34^. This suggests that interstitial loops are mostly present in our sample as well. The results of extensive dislocation loop analyses are summarized in^41^ and showed a marked difference between ion and neutron irradiation results regarding average size and number density. In addition, different irradiation conditions and different W to Re transmutation rates hamper a direct comparison of microstructure from different irradiation campaign.

The areas of about 10–20 nm around the precipitates are almost free of dislocation loops or black dots (Fig. 3). Their formation can be affected by the vicinity of precipitates, which can serve as sinks for vacancies and interstitials, and by increased local Re and Os concentrations. Previous experimental work showed that the occurrence of loops and voids depends on the Re content of the examined alloy^42–44^. Both voids and loops show a lower number density in the W-3%Re alloy compared to the pure W^42^ and were not detected in the W-25%Re alloy irradiated at temperatures ranging from 373 to 760 °C^43^. Several studies have shown that this is related to a higher binding energy of solute atoms (Re and Os) at vacancies and self-interstitial atoms (SIA) in W. The formation of Re–W or W–Os dumbbells significantly slows down their diffusion and thus increases the probability of recombination within the cascade^45–47^. The change from the one-dimensional motion of the SIAs in pure W to the significantly slower three-dimensional motion of the interstitial atoms associated with Re or Os is proposed as the decisive factor for this effect. This three-dimensional movement was made possible by the low rotational energy barrier of the mixed dumbbell compare to the pure W^45^. SIAs with one-dimensional motion can encounter vacancies only along their one-dimensional migration line, and herewith they have a greater probability of escaping from a cascade displacement influenced region without recombination compared with those with 3D motion. These SIAs can further form interstitial clusters and loops. It can be suggested that the Re concentration in the layer of 10–20 nm around the precipitates is high enough to effectively prevent the local formation of the loops.

The radiation induced migration of Re and Os atoms leads consequently to the formation of precipitates containing of σ- and χ-phases. The mechanism of association of solute atoms with defects and their diffusion is considered the most appropriate to explain radiation-induced precipitation in tungsten^45–47^. Both the vacancies and the SIA are able to drag the solute atom, but the contribution of the SIAs to the aggregation of the solute atoms is much greater than the vacancies^45^. It was shown that such solutes as Re or OS are much more strongly bound to interstitial sites than to voids. Interstitial solute complexes were found to be very effective in enriching solutes at sinks, such as voids or already formed precipitates. The enrichment of Re and Os around voids and the formation of precipitates is experimentally validated in the present work.

Neutron irradiation induced void formation is the main damage process in W. The presence of voids was reported in every specimen irradiated at temperatures exceeding 500°C^35,42^. The formation starts at slightly higher irradiation doses as the dislocation loop formation, i.e. 0.15 dpa^42^. Increasing the dose increases the average void size and decreases their number density. Despite the fact that the voids were formed under irradiation conditions that varied over a wide range, their morphology is remarkably similar. The average size typically varies from 2 to 8 nm and number density is mostly in ~ n 10^21^ m^−3^ range. The average void size of 31 nm and the number density 4 10^20^ m^−3^ reported in the present work for the grain interior (Table 3) are significantly different from those previously reported^4,6,42,48,49^. The microstructure of the material examined is in some degree unique. This means that such a large average size of voids and such low number density has not been observed before. However, the total void swelling was estimated to be ~ 0.04%, a value comparable to other results on neutron irradiated tungsten. The voids found at the temperatures ranging from 700 to 900 °C and doses exceeding 1.0 dpa are always smaller than 10 nm, with an average size ranging from 3 to 7 nm. The corresponding number density of voids in the same temperature and dose range lies between 2 10^21^–1 10^22^ m^−3^. Larger number densities, i.e. larger than 1 10^23^ m^−3^, only occur for irradiation temperatures below 760 °C at low doses^34^. The results summarized from different publications exhibit the tendency that the average void size increases from 4 to 7 nm and decrease in number density for doses exceeding 1.0 dpa, while the temperature influence on both parameters is statistically insignificant^50^. The voids formed at similar conditions (T_irr_ = 900 °C, 1.54 dpa) are considerably smaller in size and have an order of magnitude higher number density than the voids reported in current work^6^. Thus, our results are not in line with previously published data and the difference is larger than that being expected if only damage and temperature are being considered.

We suggest that such differences in void morphologies could be explained by microstructural responses to neutron irradiation in different reactors. It was reported previously that the damage dose influences the void morphology to a significantly higher degree than the irradiation temperature. For example the presence of Re had a strong influence on the size and distribution of voids^4,42^. The accumulation of Re in W during neutron irradiation restrains their nucleation and growth. Moreover, the presence of Re and Os inside irradiated W can inhibit the growth of radiation-induced defects, whereas Os plays even more significant role than Re^4,51^. Thus, the concentration of transmutation products depends not only on irradiation temperature and dose, but also on the transmutation rates, which themselves depend on the actual neutron spectrum.

When analyzing the results of W irradiation in different reactors, significant differences in the size and distribution of the voids can be found. For example, voids in pure W irradiated in the JOYO fast reactor show a number density in the 10^23^ m^−3^ range. Fukuda et al.^31^ shows for example that their number density in pure W irradiated at 537 °C comprises 1.9 10^23^ m^−3^. A similar values of 1.2 10^23^ m^−3^ (750 °C/1.54dpa) and 5 10^23^ m^−3^ (538/0.9dpa) was reported by Tanno et al.^11,52^. The formation of void lattice with 20 nm spacing in the damage dose from 0.40 to 1.54 dpa at 538 °C and 750 °C W in pure W was reported by ref.^43,51^. In contrast to these works, the formation of void lattice was not observed in the present study. The neutron spectrum in the JOYO reactor has a low cross-section for the transmutation of W and thus the changes in the chemical composition after irradiation are negligible..

Both the HFIR and JMTR reactors provide a mixed neutron spectrum, which leads to an increased production of transmutation elements and consequently to a decrease of number density of voids by a factor of 10–150. Typically, the number density of voids in W irradiated in the range of 724 °C to 764 °C varied from 7 10^20^ m^−3^ to 1 10^22^ m^−3^^34^. The number density of voids in W–Re-alloys irradiated under similar conditions does not depend on the reactor type and is in the identical range from 3 10^20^ m^−3^ to 6 10^21^ m^−3^^52^. According to Herschitz and Seidman^53^ the growth of voids in W–Re alloys is affected by the recombination of point defects, i.e. vacancies and interstitials at a biased sink, e.g. dislocations. A similar argumentation is also valid for the generation of precipitates. They also claim that coherent precipitates in W–Re alloys are only generated if they are located close to damage cascades by primary knock-on atoms.

Precipitates containing Re and Os are also formed in pure W irradiated in HFIR since the nuclear transmutation rate in HFIR is higher than in sodium-cooled reactors (JOYO) due to the high flux of thermal neutrons. Material analysed in our work shows the presence of 2% Re and 0.2% Os after irradiation to 1.25 dpa. The transmutation rate of ~ 1.6%Re/dpa is similar to that calculated for HFIR reactors by Greenwood and Garner^54^. The formation of precipitates with defined stoichiometric composition and crystalline structure starts when the irradiation temperatures exceed 500 °C and leads to the formation of σ-WRe_2_ and χ-WRe_3_ phases^7,52^. In particular, it was reported that the χ-phase has a needle-like shape and the σ-phase has a spherical shape^11,33^.

Our investigations show the formation of Os-based σ- and χ-phases located in the center of the Re- and Os-rich clouds (see Figs. 4 and 5). The similar clouds also form around all voids, are not clearly recognizable in standard TEM or STEM images and can only be visualized in STEM-EDX element maps (see e.g. Figure 4). Recently formation of clouds with a diameter of about ~ 30 nm has been reported in several publications^3,5,6,50,55^. The value is twice as small as the average diameter of 60–80 nm measured in our work. The clouds occur probably in early irradiation stages and serve as precursors for the later formation of σ-WOs_2_ and the χ-WOs_3_ precipitates when the local concentration of Os has reached a certain value through the further transmutation of Re. The existence of the σ-WOs_2_ phase in the neutron irradiated W–Os alloy was reported by Tanno, T. et al.^43^, whereas the existence of χ-WOs_3_ phases was not reported up to now. Here, we present the first experimental evidence of this phase. However, for an enhanced understanding of the precipitation behavior more experimental data would be desirable.

The binary phase diagrams of W–Re and W–Os indicate the presence of a σ-type phase at elevated temperatures. In case of W–Os this phase extends from about 65 at% to about 80 at% W for temperatures ranging from 1000 to 3000 °C. The existence of χ-WOs_3_ phase was not reported since the phase diagram was not explored for all concentration ranges and temperatures. In case of σ-WRe_2_ the situation is more complicated: above 2000 °C the σ-phase is present in the range of 30 at% to 56 at% W, whereas below the range of existence shifts from 30 at% to about 35 at% at 1600 °C. In addition, the χ-WOs_3_ phase exists up to about 2100 °C in the W concentration range of 26–28 at%. In neutron-irradiated material, however, due to the irradiation-enhanced diffusion of impurities, secondary phases are not necessarily formed in accordance with the phase equilibrium rules.

Figures 7 and 8 clearly illustrate that there is a textural (epitaxial) relationship between the W-matrix and both types of precipitates. However, as can be seen in Fig. 9, the structure of the precipitation is not always well extinct. (Semi-)coherent precipitates were also observed by atom-probe measurements in W–Re alloys^53^. In this case, W–Re alloys with similar precipitation behavior as in the present case were already studied by several groups^11,33,43,44,56^, who reported circular-shaped σ-type and platelet-shaped χ-type precipitates. This agrees also with the binary W–Re phase diagram. However, in our case pure W was subjected to neutron irradiation. The available literature is more limited than for the W–Re alloys^8,10,50^. However, recently published results of neutron irradiated pure W show a similar microstructure as our sample for similar neutron irradiation conditions, respectively^50,55^. The crystallographic structure of the intragranular precipitates is still under debate at present time. On one hand, Katoh et al.^50^ claim that the platelet-shaped precipitates found in samples irradiated at elevated temperatures and doses around one dpa are inconsistent with the σ- and the χ-type phase. Edmondson et al.^55^ on the other hand come to the conclusion that the needle-like precipitates are of the σ-type. Moreover, they found a clear relationship of the W matrix with the precipitates, i.e. the precipitates lie along the (011) planes. They attributed this behavior to the dislocation loops originating from collision cascades, which act as trap for diffusing Os and Re atoms thereby seeding the precipitates. In contrast to Katoh et al.^50^ we found that both types of precipitates (σ- and the χ-type) are present within our sample. We found that out-of-plane the σ-phase [110] axis is oriented along the [110] of W, whereas in-plane the orientation relation is (1–10)_W_ || (1–12)_σ_ and (002)_W_ || (−441)_σ_. Whether the precipitate has a rod or needle shape or another one is not evident from Fig. 7. In case of the χ-type precipitates the orientation relationship is more complicated than for the σ-type precipitates: Out-of-plane the W [110] direction is parallel to the [214] direction of the χ-phase, whereas in-plane it is (1–10)_W_ || (2–40)_χ_, (002)_W_ || (−5–32)_χ_. To our knowledge, such orientation relationships have not been reported before for this metallic system. However, in case of the χ-type phase the orientation relation is not the same for all as discussed in the following paragraph.

Katoh et al.^50^ found that some precipitates are associated with a void and some are not, which is in accordance with our observations. Furthermore, we found that the voids are often facetted with the majority of the facets having a {110}-type orientation. This can be explained by considering the surface energies of W, which are lowest for {110}-type surfaces^57^. If voids are facetted and have a precipitate attached, the precipitate grows out of a {110}-type surface. This might be attributed to the origin of both which is related to damage cascades. Some of the W–Os precipitates were found to be inclined with respect to the matrix lattice (see for example Fig. 9). This behavior was also observed by Edmondson et al.^55^. Since the statistics of analyzed precipitates is limited using high-resolution phase contrast images, tKD or EBSD-like orientation mapping in the TEM can improve the statistics significantly regarding the textural relationship between matrix and precipitates.

GBs play an important role in the formation and coarsening of radiation-induced defects (Figs. 1b, 6). They act as sinks for all kinds of point defects, i.e. vacancies and interstitials as well as in our case Re and Os atoms, resulting in the formation of a 200 nm wide void- and precipitate-free zone (called the denuded zone) adjacent to the GBs (see Fig. 1b, 6). Next to the denuded zone, a 300 nm wide void peak zone has formed, in which the voids have a reduced average size, while the number density of them is twice as high as in the grain interior (see Table 3). The formation of larger χ- and σ-precipitates in the zone also indicates a higher concentration of Re and Os compared to the grain interior (Fig. 6). According to Fukuda et al.^33^, the higher diffusivity of SIA s in comparison to that of vacancies promotes the formation of a void-peak zone in the immediate vicinity of the denuded zone.

The Re and Os concentrations show a typical "W-shaped" profile across the GB, which thickness corresponds to the denuded zone (Fig. 6e,f). The thickness of the Re-rich layer at the GB is 30 nm, while Os is 22 nm. The low thickness of the Os-rich layer compared to Re-rich layer leads to an increased local Os/Re ratio of ~ 0.2. This is the precursor to the formation of Os-rich precipitates inside a much larger Re-rich clouds^49^. The Os/Re ratio at the GB was estimated to reach ~ 0.15, while this ratio in the sample average is calculated to be 0.1. It should be noted that the quantification of EDX measurements shown here was performed near the sensitivity limit of the EDX method (0.1–0.25 wt%), where background subtraction plays an essential role for the results. On the other hand, the EDX lines of W, Re and Os are close to each other, so the background subtraction is possible with a reasonable error. These two factors cause a measurement error of up to 50% in the Re and Os concentration values.

Next to the void peak zone we observed the formation of a second denuded zone of about ~ 500 nm wide with an obviously lower number density and size of voids and Os-rich precipitates if compared to the grain interior (Table 3). In this area especially Os- and Re-rich "clouds" are dominating structures. This indicates that the formation of σ- and χ-phases occurs only in "clouds" with sufficient Re and Os concentration, obviously reached inside grains or void denuded zones. The low local concentration of Re and Os suppresses their formation and growth. The influence of the GB on the distribution of transmutation products and the formation of radiation-induced defects has already been reported in the past^3,6,33,49^. However, the width of the denuded zone reported in these works is typically ~ 20 nm—a value that is 10 times lower than what is measured in this study.

It is known that besides Re and Os also H and He are generated in the low appm amounts by transmutation of impurities in W and its alloys^58,59^. From Be it is well-known that both elements are retained within the material in the form of gas-filled bubbles^60^. The same effect might be also valid for W, however, in an attenuated form. In W, the bulk volume plasmon is located at 25.3 eV according to our measurements, which is close to the He edge at 22 eV^61^. The additional peak located only in the void area (STEM-EELS in Fig. 10) lies in principle in the correct energy range of the He edge. However, since STEM-EDX also revealed the presence of oxygen within the void acting as dielectric medium another effect called plasmon resonance is also likely to be present. In case of nanometer-sized spherical voids this effect will generate additional intensity in the EEL spectrum located at $\sqrt{2/3}{\omega }_{B}$, where ${\omega }_{B}$ is the bulk plasmon frequency^62^. In case of W this evaluates to 20.7 eV, which fits better than the He edge to the observed void spectrum. If the voids contain pressurized He gas, a blue shift of the He-K edge towards the W bulk plasmon can be expected hampering the He detection in this particular case. Atom-probe measurements by Herschitz and Seidman^53^ did not reveal any He within voids, however, they found evidence of He associated with precipitates. The question of the He presence in the voids remains still open.

In general, experimental data collected in recent years shows that the microstructure of neutron-irradiated W depends on the temperature and damage dose, as well as on the concentration of transmutation products. For the latter one the applied neutron spectrum is of great importance, which itself is different for each reactor type or shielding factor, e.g. position of the sample in the irradiation rig and of the rig itself in the reactor core. Our analyses form the basis for a detailed understanding of the microstructural development of W under neutron irradiation. However, considerably more research is needed to uncover the structural effects of a true fusion neutron spectrum.

## Conclusions

This paper presents the results of extensive microstructural analyses of W samples neutron irradiated at 800 °C to ~ 1.0 dpa. The formation of dislocation loops, voids and precipitates consisting of Re–Os σ- and χ-phases was detected and characterized in detail. The results of the study can be summarized as follows:

- The dislocation loops have an average size of 5 nm and number density of 5 10^23^ m^–3^. About 95% of the loops have a *b*½ < 111 > Burgers vector. These data are comparable with the data of W irradiated in the HFIR.
- The voids with an average size of 31 nm and number density of 4 10^20^ m^−3^ were significantly different from the data being published in the literature. The average size is about 4–6 times larger and the number density is one order of magnitude lower than the published data for W irradiated at ~ 800 °C.
- Near GBs, we detected the formation of a ~ 200 nm wide void denuded zone and a ~ 300 nm wide void peak zone.
- The Os induced by the transmutation has formed σ-WOs_2_ and χ-WOs_3_ precipitates with spherical and needle-like shape, respectively.
- The χ-WOs_3_ phase is often associated with a void in contrast to the σ-WOs_2_.
- The coherent precipitation of both σ and χ is phases was determined. The [110] direction of σ-phase is oriented along that [110] of W, whereas in-plane the orientation relation is (1–10)_W_ || (1–12)_σ_ and (002)_W_ || (−441)_σ_. The out-of-plane the W [110] direction is parallel to the [214] direction of the χ-type precipitates, whereas in-plane it is (1–10)_W_ || (2–40)_χ_, (002)_W_ || (−5–32)_χ_.
- Formation of Re and Os rich clouds around voids and precipitates was observed and analyzed.
